# *Streptococcus mutans,* Caries and Simulation Models

**DOI:** 10.3390/nu2030290

**Published:** 2010-03-02

**Authors:** Sofia D. Forssten, Marika Björklund, Arthur C. Ouwehand

**Affiliations:** Danisco Finland, Sokeritehtaantie 20, 02460 Kantvik, Finland; Email: marika.bjorklund@danisco.com (M.B.); arthur.ouwehand@danisco.com (A.C.O.)

**Keywords:** Caries, *Streptococcus mutans*, carbohydrates, simulation, hydroxyapatite, artificial saliva

## Abstract

Dental caries and dental plaque are among the most common diseases worldwide, and are caused by a mixture of microorganisms and food debris. Specific types of acid-producing bacteria, especially *Streptococcus mutans,* colonize the dental surface and cause damage to the hard tooth structure in the presence of fermentable carbohydrates e.g., sucrose and fructose. This paper reviews the link between *S. mutans* and caries, as well as different simulation models that are available for studying caries. These models offer a valuable approach to study cariogenicity of different substrates as well as colonization of *S. mutans*.

## 1. Introduction

Dental caries is one of the most common and costly diseases in the world, and although rarely life threatening it is a major problem for health service providers. In order to decrease the prevalence of caries, an improved understanding of the role of the microorganisms in dental diseases is needed [1]. The tooth surface is covered with a biofilm–a slime layer consisting of millions of bacterial cells, salivary polymers, and food debris. Uncontrolled, this biofilm can easily reach a thickness of hundreds of cells on the surfaces of the teeth. The formed biofilm, also called plaque, provides an excellent adhesion site for the colonization and growth of many bacterial species. 

## 2. Microorganisms Present in the Mouth

The oral microbiota functions as a part of the host defense by acting as a barrier, e.g., by competition for essential nutrients and creation of unfavorable conditions to exogenous organisms that may be pathogenic to the host. Over 700 bacterial taxa have been found in the oral cavity, however they are not all present in the same mouth [2]. The composition varies in different sites in the oral cavity, with e.g., a large and more diverse bacterial load on the dorsum of the tongue. Most of these microbes are harmless, but under certain conditions some can cause oral infections like caries or periodontal disease [3]. Oral streptococci, like *Streptococcus mutans*, are associated with pyogenic and other infections in various sites including mouth, heart, joints, skin, muscle, and central nervous system [4]. 

## 3. Dental Plaque

Three steps are involved in the formation of dental plaque; First salivary molecules are adsorbed to the enamel as soon as a tooth has been cleaned. Hence the enamel is coated with a complex mixture of components that include glycoproteins, acidic proline-rich proteins, mucins, bacterial cell debris, exoproducts, and sialic acid. The second step is bacterial interaction with this acquired pellicle via several specific cell-to-surface interactions [5]. The biofilm formation of the primary colonizers, mainly *Streptococcus sanguis* and *Actinomyces viscosus* [6], is influenced by a number of environmental parameters, such as osmolarity, carbon source, and pH [5]. During the third step, other bacterial species like *S. mutans* adhere to the primary colonizers by cell-to-cell interactions. Subsequent bacterial growth on tooth surface leads to formation of biofilm on the teeth, also called dental plaque [5,7].

An increase in dietary carbohydrates, particularly sucrose, results in additional acid production that may exceed both the capacity of the saliva to remove acid end-products and the neutralizing power of the salivary/plaque buffer system, and results in more frequent acidification of the plaque [8]. Diet containing sucrose is one of the main reasons for the high dental caries rate in developed countries [9,10]. 

### 3.1. *S. mutans* and Caries

*S. mutans* gives its name to a group of seven closely related species collectively referred to as the mutans streptococci. The primary habitats for *S. mutans* are mouth, pharynx, and intestine [11]. Several factors, such as adherence to enamel surfaces, production of acidic metabolites, the capacity to build up glycogen reserves and the ability to synthesize extracellular polysaccharides are present in dental caries [11,12]. *S. mutans* and *Streptococcus sobrinus* have a central role in the etiology of dental caries [1,13], because these can adhere to the enamel salivary pellicle and to other plaque bacteria [6]. Mutans streptococci and lactobacilli are strong acid producers and hence cause an acidic environment creating the risk for cavities [14]. Usually, the appearance of *S. mutans* in the tooth cavities is followed by caries after 6-24 months [15]. The acidogenic *S. mutans* and *S. sobrinus* are able to form extracellular polysaccharides (EPS) in the presence of sucrose [16,17], but also from fructose and glucose. The EPS are long-chained and high molecular mass polymers [18]. The energy rich glycosidic bond between the glucose and fructose moieties supplies the free energy needed for the synthesis of EPS. Glucose homopolysaccharides are called glucans while fructose homopolysaccharides are called fructans [8,19]. Glucans are produced by glucosyltransferases (GTF) while fructans are produced by fructosyltransferases (FTF) [20]. The production of large quantities of EPSs from sucrose is an important factor of *S. mutans* cariogenicity [21]. 

### 3.2. Effect of Sucrose on EPS

Any carbohydrate that dental plaque bacteria can utilize as an energy source contributes more or less to the virulence of the microbiota and thus has a cariogenic potential. Sucrose is not only rapidly fermented to acidic end products, but it is also the only dietary carbohydrate that can be transformed into EPS in the plaque. Thus, it is considered to be the most cariogenic carbohydrate in the human diet [8]. The carbohydrate composition of EPS may vary depending on the growth conditions, important variables being pH, temperature and nitrogen source. EPS production is mainly growth associated; therefore the greatest rate of production occurs under conditions optimal for growth [18]. The brain-heart infusion (BHI) medium has been shown to be the most effective regarding production of EPS [20]. 

How EPSs affect the progress of dental caries has been described as follows [21]: (1) polysaccharides provide reserve of substrates; (2) EPSs aid adherence; (3) water-insoluble EPSs act as diffusion barriers thus trapping acid near the tooth surface; and (4) EPSs increase the plaque thickness and thus acid retention time. Nevertheless, it was also observed that neither acid nor carbohydrate diffusion is much affected by EPS content [21]. Thus EPSs are not likely to act as diffusion barriers, and inhibition of acid and carbohydrate diffusion is not one of the important factors in the caries-promoting effect of EPSs. This was supported by a study by Hata and Mayanagi [22], who also doubted the diffusion barrier effect of EPSs. They studied the role of glucans and fructans in the diffusion of ions through cell concentrates. Their conclusion was that water-insoluble glucans provide a source of fermentable substrates. Thus, they suggest that water-insoluble glucans can enhance the cariogenic potential of EPSs by allowing greater and are more sustainable access to nutrients. Glucans act as adhesives onto the tooth surface and are hence essential for the cariogenicity of *S. mutans* [23]. In addition, glucans are essential in cell-cell and cell-surface adhesive interactions in plaque with dextran-mediating bacterial aggregation [17]. Although water-soluble glucan is the main glucan responsible for the plaque progress, mutants unable to synthesize water-insoluble glucan exhibit markedly reduced cariogenicity. Shellis and Dibdin [21] and Wiater *et al.* [17] were convinced that the main role of EPSs in cariogenicity of *S. mutans* is due to the adherence properties.

### 3.3. Prevention of Dental Plaque

The control of plaque can be achieved by mechanical oral hygiene procedures, but in many cases that is not enough. Thus, addition of antiplaque or antimicrobial agents to dental health care products has been of value. The main mechanisms by which plaque can be controlled by the use of chemotherapeutic agents are: to reduce the overall rate of accumulation of new plaque, to reduce or remove existing plaque, to inhibit only the growth of those species implicated in disease, and to inhibit the production of e.g., EPSs [24].

Xylitol is a sugar alcohol or polyol that is naturally present in the human metabolism, and can thus be safely used in dental products or as a food ingredient [25]. It has been shown that xylitol has antibacterial properties [26,27] and that systematic use of xylitol reduces the incidence of caries [25,27,28,29,30] and growth of *S. mutans* [12,27]. Xylitol has been reported to affect the synthesis of polysaccharides in *S. mutans*, which leads to a decreased bacterial adherence [31]. Another theory is that xylitol is noncariogenic since *S. mutans* is not able to ferment xylitol. Even though the effects of xylitol on caries prevention have been studied for 40 years, the detailed mechanism of action is still to be discovered. Triclosan is an organic compound with antibacterial properties that has been used in toothpaste for decades, and has been shown to have an inhibitory effect on bacterial metabolism in dental plaque and to improve gingival health [32]. Triclosan inhibits the growth of *S. mutans* by sensitizing glycolysis to acid inhibition by acting with weak-acid transmembrane proton carriers, such as fluoride [32,33,34]. However, xylitol can be considered as a safer substance to use than triclosan for the prevention of plaques, since triclosan can react with chlorine in tap water and form chloroform and is hence considered to be toxic. In addition, xylitol has received an anti-cariogenic claim approval by the European Food Safety Authority (EFSA).

### 3.4. Models for Studying Caries

As bacterial adhesion is essential for creating a pro-cariogenic micro-environment, it is important to investigate the adherence of bacteria. Common surfaces that are used in adhesion testing are hydroxyapatite (HA), dentine, microtiter wells, glass surfaces, ceramic surfaces, bovine enamel and human teeth. Quantification of adhered microbes can be done by radiolabeling, optical density or culturing after release of adhered microbes, DNA or protein quantity, attenuated total reflectance Fourier transform infrared spectroscopy or confocal scanning laser microscopy.

Haukioja and co-workers described the use of hydroxyapatite beads, coated with bovine serum albumin (BSA) or human saliva, and human saliva coated microtiter wells [35]. In general, a reasonable correlation was observed for binding by the tested lactic acid bacteria. The general high binding to both saliva and BSA may suggest a nonspecific binding mechanism.

The so-called Zürich Biofilm Model aims to create a six strain plaque biofilm on hydroxyapatite disks. The bacteria are grown for 64.5 hours and human saliva is replaced every 24 hours. The model can be used to assess anti-plaque agents [36]. More dynamic dental plaque biofilm models have been developed to facilitate investigation of single or multi-species plaque development and metabolism. A chemostatic flow cell system model was described by Herles *et al.* [37], where five species of oral bacteria were grown in an artificial saliva medium, which was pumped through six flow cells during a 72-hour experimental period. Each cell contained two types of surfaces on which plaque was formed; hydroxyapatite and germanium. With this model, it was possible to follow the species specific composition of plaque organisms and a time dependent build up of plaque that could be reduced with triclosan compared to water [37]. 

A microbial-based caries model where human teeth are fixed on a rotating mount within a reaction chamber has been described [38]. The cariogenic environment was created by inoculation with *S. mutans* combined with a continuously dripping supply of sequentially trypticase soy broth, artificial saliva and sucrose solution, onto the teeth. This model allows the simultaneous production of primary and secondary caries-like enamel lesions. 

Another plaque model called the multiple artificial mouth (MAM) system has also been described [39]. This model consists of a plaque growth chamber where plaques are grown on pellicle-coated cover slips, and the addition of reagents and growth conditions for each plaque can be independently controlled. 

**Figure 1:**
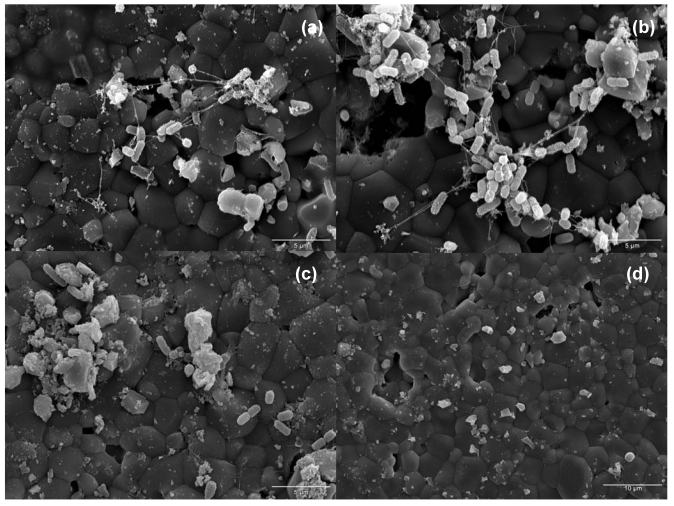
Attachment of *Streptococcus mutans* Ingbritt to the HA disk by SEM in the presence of **(a) **artificial saliva, **(b) **artificial saliva containing 1% sucrose, **(c) **artificial saliva containing **1**% sucrose and 4% xylitol. The surface of a clean (no bacteria attached) HA disk is represented in **(d)**.

The Enteromix® caries simulator is a continuous flow system that consists of a chamber system of 16 bottles with a continuous flow of artificial saliva that simulates the oral cavity. HA discs are included in the system to mimic the teeth and to offer adhesive surfaces for *S. mutans*. A bacterial suspension can be inoculated into the culture vessels. The content of the bottles is well stirred to approximate to the ideal of complete mixing. Thus microbial growth occurs at a constant rate and in a constant environment, under steady-state conditions in a continuous culture. Carbohydrates can be supplied into the culture vessels along with the artificial saliva from the medium supply bottles during the simulation. The bacteria attached to the HA disk can be visualized by Scanning Electron Microscopy (SEM) and quantified by DNA extraction and qPCR. The attachment of *S. mutans* to HA under different treatments is visualized in Figure 1.

With the *in vitro* models it is however not possible to mimic the biological conditions in the oral cavity and hence, the use of animal models are also valuable. With animal models it is possible to study oral infections like gingival lesions in addition to caries [40], since the simulation is then under biological conditions [41,42].

## 4. Conclusions

The principle cause of dental caries is well understood; the consumption of easily fermentable carbohydrates (in practice usually sucrose) stimulates the growth of oral microbes, most notably *S. mutans*. Due to the absence of oxygen and the species’ general fermentative metabolism, growth on sucrose leads to the formation of organic acids. Even though the produced amounts of acid are small, they are produced very locally and reduce the pH in the bacterium’s microenvironment. Since *S. mutans* is able to directly adhere to the tooth’s HA matrix, the pH of the tooth surface may be easily reduced to below the critical pH of HA demineralization (pH 5.5).

Caries models have been developed to provide a practical and ethical manner to investigate cariogenicity in defined environments. They have proven to be of great importance in evaluating bacterial growth and metabolism, more advanced systems being able to predict also pathogenicity. Simple *in vitro* testing measures bacterial growth and acid formation in a stationary medium, often in the presence of commonly anti-cariogenic substances. To create an environment that mimics the growth conditions in the mouth, a continuous flow system is more appropriate than a stationary system. Since different surfaces are used and the adhesion varies between surfaces and surface coatings, such as coating saliva composition or proteins [35], it is difficult to compare methods that use different adhesion surfaces. The various caries model systems combine several of the functions described to provide a setting that mimics the *in vivo* oral niches and habitats. Many of the models are quite simple and investigate the properties of e.g., only one or a couple of strains at a time. The Enteromix® caries simulator has a continuous flow system to mimic the rinsing effect of saliva in order to study the adhesion and proliferation of the bacteria. The continuous flow mimics the actual conditions of salivary flow, adding fresh growth medium and simultaneously diluting the growing bacteria. The addition of xylitol to sucrose-supplemented artificial saliva reduced the colonization and EPS formation of *S. mutans*, and hence the cariostatic effect of xylitol is verified by this simulation system, suggesting that not the mere replacement of a fermentable carbohydrate by a non-fermentable one is the underlying mechanism of xylitol’s anti-cariogenic action. The weakened adhesion of the bacterium onto teeth surfaces is significant for the prevention of caries. 

In conclusion, dental caries is one of the most common and costly diseases in the world. Thus, strategies to reduce the risk for dental caries are important. The strategies usually involve decreasing the growth or activity of *S. mutans.* The simulation models are valuable alternatives to animal model testing. In addition, the experimental conditions can be more efficiently controlled than animal models. Hence, the simulation models are of great value in predicting potential caries-preventive strategies. 
